# Small Extracellular Vesicles Secreted by Cisplatin-Resistant Neuroblastoma Cells Increase Lactate Secretion and Alter Metabolic Pathways in Primary Human Umbilical Vein Endothelial Cells (HUVECs)

**DOI:** 10.3390/jpm15120584

**Published:** 2025-12-01

**Authors:** Thomas Frawley, Lin Ma, Muhammad Zainul Arifin, Dan Wu, Alysia Scott, Brenton Cavanagh, Donal F. O’Shea, Vadim Zhernovkov, Mi Liu, Marco P. Monopoli, Olga Piskareva

**Affiliations:** 1 Cancer Bioengineering Group, Department of Anatomy and Regenerative Medicine, RCSI University of Medicine and Health Sciences, 123 St. Stephen’s Green, D02 YN77 Dublin, Ireland; 2 Tissue Engineering Research Group (TERG), Department of Anatomy and Regenerative Medicine, RCSI University of Medicine and Health Sciences, 123 St. Stephen’s Green, D02 YN77 Dublin, Ireland; 3 School of Pharmacy and Biomolecular Sciences, RCSI University of Medicine and Health Sciences, 123 St. Stephen’s Green, D02 YN77 Dublin, Ireland; 4 Kunshan Hospital of Traditional Chinese Medicine, Kunshan 215300, China; 5 College of Pharmaceutical Sciences, Soochow University, Suzhou 215123, China; 6 Systems Biology Ireland, School of Medicine, University College Dublin, Temple Street, D04 C1P1 Dublin, Ireland; 7 Department of Chemistry, RCSI University of Medicine and Health Sciences, 123 St. Stephen’s Green, D02 YN77 Dublin, Ireland; 8 Cellular and Molecular Imaging Core, RCSI University of Medicine and Health Sciences, D02 YN77 Dublin, Ireland; 9 Advanced Materials and Bioengineering Research Centre (AMBER), RCSI (D02 YN77) and TCD (D02 CP49), Dublin, Ireland

**Keywords:** neuroblastoma, cisplatin resistance, extracellular vesicles, proteomics, Gene Set Enrichment Analysis (GSEA), metabolism, exosomal biomarkers, liquid biopsies

## Abstract

**Background**: Chemoresistance, particularly to cisplatin, remains a significant challenge in treating high-risk neuroblastoma, resulting in a mere 20% five-year overall survival rate. Tumour-derived small extracellular vesicles (sEVs) have been implicated in cancer progression by promoting angiogenesis, invasion, and proliferation in recipient cells. This study investigated alterations in the protein cargo of sEVs secreted by cisplatin-sensitive and resistant neuroblastoma cells and their impact on reprogramming non-cancerous recipient cells. **Methods**: sEVs from cisplatin-resistant (KellyCis83) and its cisplatin-sensitive parental cell line (Kelly) were isolated and characterised, followed by proteomic profiling and Gene Set Enrichment Analysis. Functional assays using human umbilical vein endothelial cells (HUVECs) evaluated the effects of sEVs on proliferation, migration, tube formation, and metabolism. The clinical relevance of the shortlisted sEV glycolytic proteins was evaluated using the R2 Genomics Analysis and Visualisation Platform. **Results**: Proteomic analysis revealed dysregulated metabolic pathways in KellyCis83 sEVs. While Kelly’s and KellyCis83’s sEV-induced aerobic glycolytic rates were similar, oxidative phosphorylation (OXPHOS) was significantly reduced in HUVECs treated with Kelly’s sEVs compared to KellyCis83’s sEVs, which might have been due to an altered balance of glycolytic enzymes in sEVs. Under angiogenic-factor-deprived conditions, the uptake of sEVs by HUVECs reduced their proliferation and increased anchorage-dependent differentiation. Our study demonstrated the enrichment of the MYCN oncogene and clinically relevant glycolytic proteins in neuroblastoma cell-derived sEVs. **Conclusions**: This study reports a potential mechanism by which sEVs derived from cisplatin-resistant neuroblastoma cells modulate endothelial cell function through alterations in metabolic pathways and provides an opportunity to explore exosomal MYCN and glycolytic proteins as circulating biomarkers for progression and treatment response signatures, using less invasive methods and enabling personalised treatment approaches for neuroblastoma patients.

## 1. Introduction

Neuroblastoma (NB) is an aggressive paediatric cancer originating from the sympathetic nervous system, primarily affecting children under five years old [1]. Its aggressiveness is linked to several molecular features, including chromosomal abnormalities 1p, 3p, 14q, and 11q; unbalanced gain of 1q, 11p, and 17q; and MYCN amplification (a transcription factor belonging to the MYC proto-oncogene family), which occurs in 20–25% of patients and is associated with high-risk disease [2,3]. The first line of treatment often includes a combination of cisplatin, vincristine, carboplatin, etoposide, and cyclophosphamide. Then, high-risk patients undergo surgery, radiation, and myeloablative treatments using escalating chemotherapy by bone marrow infusion. High-risk NB patients face a grim prognosis despite intensive multimodal treatment involving chemotherapy combinations, surgery, radiation, and myeloablative therapies [4]. The five-year overall survival rate for this cohort is a mere 20%, mainly due to the development of chemoresistance.

Acquired chemoresistance profoundly alters cancer cell signalling at multiple levels—genomic, epigenomic, transcriptomic, proteomic, and metabolomic. These alterations significantly impact cell-to-cell interactions, including the biogenesis, content, and biological activity of extracellular vesicles (EVs). EVs, particularly small EVs (sEVs) (30–150 nm), play a crucial role in intercellular communication in both normal and pathological conditions [5,6]. sEVs not only transfer bioactive molecules, including oncoproteins and nucleic acids, but they also promote tumour growth and metastasis through various mechanisms, such as angiogenesis, invasion, and proliferation in recipient cells [7,8,9].

Recent studies have revealed that sEVs from drug-resistant cancer cells can directly transfer chemoresistance to drug-sensitive cells with multiple different chemotherapy agents across various cancer types, such as breast, ovarian, leukaemia, prostate, colorectal, and pancreatic cancer [7]. In NB, sEVs from *MYCN*-amplified cells have been shown to promote doxorubicin resistance in non-*MYCN*-amplified cells, which was reversed with shRNA knockdown of *MYCN* expression [10]. Furthermore, NB-derived sEVs can indirectly promote chemoresistance by modulating the tumour microenvironment (TME). For example, Challagundla et al. demonstrated that NB-derived sEVs can induce human monocytes to release sEVs containing miR-155, which, upon uptake in NB cells, conferred cisplatin resistance [11]. In a more recent study, doxorubicin-treated NB cells were found to secrete sEVs that promoted pre-metastatic niche formation and increased metastasis in NB cell line mouse models [12].

A key aspect of sEV-mediated effects is their ability to induce metabolic reprogramming, such as aerobic glycolysis and reduced oxidative phosphorylation in recipient cells, promoting the Warburg effect [8]. Several studies demonstrated that sEV-driven metabolic changes promote angiogenesis in acute myeloid leukaemia (AML), colorectal cancer, and head and neck cancer using an HUVEC tube formation assay via different mechanisms such as miR-221-3p transfer in AML, SRSF3-mediated VEGF upregulation in colorectal cancer, and MMP-9 enrichment in head and neck cancer [13,14,15,16]. Tumour growth and propagation require oxygen and nutrient supply, metabolites, and an effective way to remove waste products, which can be facilitated through newly developed vasculature. However, nothing is known about how the metabolic reprogramming by sEVs can foster angiogenesis in NB.

Our hypothesis posits that acquired cisplatin resistance in NB alters the proteome and function of NB-derived sEVs, leading to enhanced metabolic reprogramming, angiogenesis, and propagation of a chemoresistant phenotype. To test this hypothesis, we employed a well-characterised in vitro model—cisplatin-sensitive Kelly and cisplatin-resistant KellyCis83 NB cell lines [17,18,19]. The KellyCis83 cell line was established by gradually exposing parental Kelly cells to increasing concentrations of cisplatin over 6 months, reaching a final concentration of 83 µM. This method effectively simulates the gradual acquisition of drug resistance observed in clinical settings.

Comprehensive genomic and phenotypic analyses have demonstrated that KellyCis83 is distinct from its parental line while retaining key features of aggressive NB. Array comparative genomic hybridisation (aCGH) revealed that KellyCis83 has acquired 12 additional chromosomal aberrations, including 9 gains, 2 losses, and 1 homozygous deletion. The cell line has been thoroughly profiled using mass spectrometry, proliferation assays, and toxicity assays, providing detailed insights into its cellular characteristics [17]. Thus, confirming KellyCis83 cell line is both phenotypically and genomically distinct from the parental Kelly cell line, while maintaining *MYCN* amplification, a hallmark of high-risk NB, and ensuring its relevance as a model for aggressive disease.

This well-defined model allows us to identify the effects of acquired cisplatin resistance on sEV composition and function, providing a robust foundation for our investigation. By utilising the Kelly/KellyCis83 pair, we can directly compare sEVs from cisplatin-sensitive and cisplatin-resistant cells, offering valuable insights into how drug resistance influences intercellular communication via sEVs.

Our study focuses on how acquired cisplatin resistance alters the protein cargo of sEVs secreted by NB cells and how these changes reprogram the functions of non-cancerous recipient cells to promote a pro-tumorigenic phenotype. This approach has the potential to uncover novel mechanisms of chemoresistance propagation and identify new circulating biomarkers and therapeutic targets to improve outcomes for high-risk NB patients.

In this paper, the terms ‘exosomes’ and ‘extracellular vesicles (EVs)’ are used in reference to original studies published before the updated Minimal Information for Studies of Extracellular Vesicles (MISEV) guidelines [20,21]. This terminology has been retained to reflect original characterisation methods, which differ from current standards.

## 2. Materials and Methods

### 2.1. Cell Lines

The NB cell lines SHEP-Tet-21N, Kelly and KellyCis83 were cultured in Gibco™ RPMI 1640 Medium (Gibco #21875–034, Paisley, UK) supplemented with 10% Gibco™ Foetal Bovine Serum (FBS) (Gibco #10270–106, Grand Island, NY, USA), and 1% Gibco™ Penicillin-Streptomycin (P/S) (5000 U/mL, Gibco #15140–122, Grand Island, NY, USA). SH-SY5Y cells were cultured in a 1:1 ratio of Gibco™ MEM (Gibco #11090081, Grand Island, NY, USA) no L-glutamine and Gibco™ Ham’s F-12 Nutrient Mix supplemented Gibco #P6010073, Paisley, UK) with 10% FBS and 1% P/S. Kelly (ECACC 92110411) and SH-SY5Y (ECACC 94030304) were sourced from the European Cell Culture Collection. KellyCis83 cells, cisplatin-resistant derivatives of Kelly, were extensively characterised by array comparative genomic hybridisation (aCGH), mass spectrometry, and proliferation and toxicity assays [17]. KellyCis83 has 12 more chromosomal aberrations (9 gains, 2 losses, 1 homozygous deletion) compared to the parental Kelly.

HUVECs are an adherent cell line pooled from the umbilical cord tissue of three newborn patients (PromoCell (HUVEC-c pooled, Growth Medium 2, order number: C-12208, lot number: 447Z015, PromoCell, Heidelberg, Germany)). HUVECs were cultured in Endothelial Cell Growth Medium 2 (PromoCell, Cat no. C-22111, Heidelberg, Germany) and supplemented with a growth factor cocktail (Table 1). All cell lines were authenticated by DNA profiling before use.

The doxycycline-inducible SHEP-Tet-21N neuroblastoma cell line allows for controlled expression/repression of MYCN. SHEP-Tet-21N cells were obtained from Dr Louis Chesler with the permission of Prof. Manfred Swab [22]. To experimentally assess the relationship between MYCN overexpression and LDHA, the doxycycline-inducible SHEP-Tet-21N neuroblastoma cell line was used. SHEP-Tet21N cells were grown in the presence and absence of doxycycline to generate samples with and without MYCN expression, designated as MYCN-DOWN and MYCN-UP. For MYCN-overexpression samples (MYCN-UP), SHEP-Tet21N cells were grown by conventional cell culture in RPMI-1640 media supplemented with 10% FBS and 1% P/S. For MYCN-repressed samples (MYCN-DOWN), 25 ng/mL doxycycline (Sigma #17086-28-1, Saint Louis, MO, USA) was added to the culture media, and the cells were grown in this medium for 6 days.

### 2.2. Cell Culture and Conditioned Media Harvest from CELLine™ AD1000 Bioreactor

For sEV production, 2.5 × 10^7^ Kelly and KellyCis83 cells were cultured in CELLine™ AD1000 bioreactor flasks (DWK Life Sciences, Cat #WCL1000AD-3, Stoke-on-Trent, UK). The AD1000 flasks consist of two compartments, media and cellular, separated by a 10 kDa semipermeable membrane. Cells were cultured in 15 mL of RPMI 1640 supplemented with 10% Gibco™ FBS, exosome-depleted (Cat #A2720801, Grand Island, NY, USA) and 1% Gibco™ Antibiotic-Antimycotic (100X) (Cat #15240062, Grand Island, NY, USA). In the media compartment, 500 mL of RPMI 1640 supplemented with 10% FBS and 1% P/S was added. Conditioned media were harvested after 6 days and centrifuged at 1200 rpm (Eppendorf, Model No. 5804, Hamburg, Germany) for 2 min to remove any cells, before centrifugation at 2000× *g* (Sorval ST40R with a TX-1000 rotor, Langenselbold, Germany) for 30 min at 4 °C, to remove large EVs. Clarified conditioned media were stored at −80 °C before sEV isolation.

Sub-culturing of cells cultured in bioreactors involved removal of spent media, washing of the cellular compartment twice with 11 mL phosphate-buffered saline (PBS), followed by enzymatic detachment with 13 mL of Gibco™ Trypsin-EDTA (0.25%) for 5–10 min. Collected cells were centrifuged at 1200 rpm for 3 min (Eppendorf, Model No. 5804). The cellular compartment was washed with 11 mL of PBS before reintroduction of 2.5 × 10^7^ cells into the cellular compartment with 13 mL of complete media. The rubber port, which provides access to the cellular compartment, was sterilised regularly to minimise the chance of infection during the sub-culturing process with 70% alcohol wipes. The trypan blue exclusion assay was regularly performed to determine the cellular viability of passaged cells.

### 2.3. sEV Isolation

sEVs were isolated by differential centrifugation in 36 mL polypropylene thin-walled tubes (Thermo Scientific™, Cat #03141). Conditioned media was centrifuged at 16,000× *g* (Sorval WX Ultra with a Surespin 630 rotor, Langenselbold, Germany) for 30 min at 4 °C. The supernatant was collected and centrifuged at 110,000× *g* for 90 min to pellet sEVs. The sEV pellet was resuspended in 30 mL 0.2 µm filtered PBS and centrifuged again at 110,000× *g* for 90 min. The final sEV pellet was resuspended in either PBS or RIPA buffer, depending on downstream analysis, and stored at 4 °C to be analysed within 3 days. sEV isolates resuspended in RIPA for proteomics were stored at −20 °C prior to analysis. When sEVs were co-cultured with cells in culture, they were filtered with a 0.45 µm Millex-HV syringe filter unit (Millipore, Cat #SLHV013SL, Darmstadt, Germany) to remove any biological contaminants introduced in the isolation process.

### 2.4. Nanoparticle Tracking Assay (NTA)

Pelleted sEVs were diluted in 1 mL of 0.2 µm filtered PBS to yield a concentration of 40–80 particles per frame. Each sample was analysed at 20–25 °C with a blue 488 nm laser and a camera level of 14 using the Nanosight NS300 (Malvern Instruments, Great Malvern, UK) NTA platform. A sCMOS camera with a slider shutter of 1000, a slider gain of 400, and a frame rate of 25 fps was used to capture three 60 s videos of suspended sEVs. Data were analysed using the NanoSight NTA 3.2 software version Dev Build 3.2.16. The detection threshold was set to five, and the maximum jump mode, maximum jump distance, and minimum track length were all set to auto.

### 2.5. Transmission Electron Microscopy (TEM)

sEV samples were air-dried on top of formvar/silicon monoxide 200 mesh copper grids (Ted Pella, Cat #01830, Redding, CA, USA) for 10 min. A negative stain of a 1:4 dilution of uranyl acetate alternative (Ted Pella, Cat #19485, Redding, CA, USA) was applied for 1 min and imaged on a Hitachi H7650 (Hitachi, Tokyo, Japan) at 100 kV.

### 2.6. ExoView Platform

Samples were analysed on the ExoView platform with the ExoView Tetraspanin Kit (NanoView Biosciences, Brighton, MA, USA). Each sample was diluted 1:1 in a manufacturer-supplied incubation solution and incubated overnight at room temperature on ExoView Tetraspanin Chips. The chips were washed three times in solution A prior to incubation with fluorescent tetraspanin antibodies. The tetraspanin fluorescent antibodies consisted of anti-CD9 CF488, anti-CD81 CF555, and anti-CD63 CF647. Antibodies were diluted 1:500 as per the manufacturer’s instructions and incubated on chips for one hour at room temperature. The chips were then washed in kit-supplied buffers, dried, and imaged by the ExoView R100 using nScan v2.7. Data were analysed using NanoViewer v2.7. Fluorescent cut-offs were set relative to the murine IgG (mIgG) control.

### 2.7. Western Blot

sEV and cells were lysed in RIPA buffer and Halt™ protease inhibitor cocktail (100X) (Thermo Scientific™, Cat #78430, Rockford, IL, USA) followed by quantification of protein concentrations of sEV and cell lysates by the Micro BCA™ (mBCA) Protein Assay Kit (ThermoFisher Scientific™, Cat #23235, Rockford, IL, USA) and Pierce™ BCA Protein Assay Kit (ThermoFisher Scientific™, Cat #23225, Rockford, IL, USA), respectively, following the manufacturer’s guidelines. Protein samples were diluted in 4X Bolt™ LDS sample buffer (Invitrogen™, Cat #B0008, Carlsbad, CA, USA) and denatured at 95 °C for 5 min. Equal concentrations of protein, between 1 and 5 µg, were separated by SDS-PAGE on Bolt™ 4–12% bis-tris plus gels (Invitrogen™, Cat #NW04120, NW04122, NW04125, Carlsbad, CA, USA), followed by the protein transfer to a nitrocellulose membrane (ThermoFisher Scientific™, Cat #88018, Rockford, IL, USA). Blots were first incubated in blocking buffer 5% BSA/TBS-T, followed by incubation with primary antibodies for 16–28 h at 4 °C with constant agitation. Primary antibodies were diluted in blocking buffer, with a dilution ranging from 1:250 to 1:2000 (Supplementary Table S1). After primary antibody incubation, each blot was washed five times with TBS-T for a total of 40–60 min. Each blot was then incubated with an appropriate secondary antibody in TBS-T; anti-mouse IgG, HRP-linked antibody (Cell Signaling Technology, Cat #7076S, Danvers, MA, USA), anti-rabbit IgG, HRP-linked antibody (Cell Signaling Technology, Cat #7074S, Danvers, MA, USA), or anti-rat (Abcam, Cat #ab6734, Cambridge, MA, USA) for 60 min at room temperature. Each blot was developed using Novex™ ECL Chemiluminescent Substrate Reagent Kit (Invitrogen™, Cat #WP20005, Carlsbad, CA, USA) and imaged on the Amersham Imager 600 (Freiburg, Germany).

### 2.8. Nuclear and Cytoplasmic Fractionation

To separate nuclear and cytoplasmic fractions from Kelly and KellyCis83 cells, we modified the protocol outlined by Schreiber et al. [23]. Cell pellets were mixed, on ice, with hypotonic cytoplasm extraction (CE) buffer.

### 2.9. Mass Spectrometry

Protein was isolated from Kelly and KellyCis83 sEV cytosolic and nuclear fractions, using RIPA buffer. Protein samples were run at 190 volts for 5 min on a Bolt™ 4–12% Bis-Tris plus gel. Tryptic digests were produced by in-gel digestion according to the protocol outlined by Shevchenko et al. [24]. Proteomics samples were analysed on a timsTOF Pro mass spectrometer (Bruker Daltonics, Bremen, Germany) coupled to a nanoElute (Bruker Daltonics, Bremen, Germany) ultra-high-pressure nanoflow liquid chromatography system (UHPnLC). The peptides were separated on a reversed-phase C18 Aurora column (25 cm × 75 μm ID, C18, 1.6 μm; IonOpticks, Australia) at a constant flow rate of 250 nL/min and an increasing acetonitrile gradient. Mobile phases were 0.1% (*v*/*v*) formic acid in water (phase A) and 0.1% (*v*/*v*) formic acid in acetonitrile (phase B). The peptides were separated using a gradient that started from 2% mobile phase B and increased linearly to 32% over 60 min. This was stepped up to 95% of mobile phase B where it was maintained for 7 min. The injection volume was 5 μL, equivalent to a loading of 250 ng per sample. The timsTOF Pro mass spectrometer was operated in positive ion polarity with TIMS (Trapped Ion Mobility Spectrometry) and PASEF (Parallel Accumulation Serial Fragmentation) modes enabled. The accumulation and ramp times for the TIMS were both set to 100 ms., with an ion mobility (1/k0) range from 0.62 to 1.46 Vs/cm. Spectra were recorded in the mass range from 100 to 1700 *m*/*z*. The precursor (MS) Intensity Threshold was set to 2500, and the precursor Target Intensity was set to 20,000. Each PASEF cycle consisted of one MS ramp for precursor detection followed by 10 PASEF MS/MS ramps, with a total cycle time of 1.16 s.

The mass spectrometry proteomics data have been deposited to the ProteomeXchange Consortium (http://proteomecentral.proteomexchange.org (accessed on 5 October 2025)) via the PRIDE partner repository [25] with the dataset identifier PXD069251 and the direct project link https://www.ebi.ac.uk/pride/archive/projects/PXD069251 (accessed on 5 October 2025).

### 2.10. Data Processing

Data were processed using MaxQuant version 1.6.15.0 and the Human UniProt database (downloaded 12 November 2019) with the following parameters: fixed mod—carbamidomethylation; variable mods—methionine oxidation, acetyl (protein N-term); trypsin/P digest enzyme; precursor mass tolerances 4.5 ppm; fragment ion mass tolerances 20 ppm; peptide FDR 1%; and protein FDR 1%. Reverse matches and potential contaminants were removed by row filtering, along with proteins identified by a single site or only one peptide. Label-free quantification (LFQ) intensities were log2-transformed and filtered for valid values. Prior to differential abundant protein identification, LFQ intensities were further filtered and imputed with PhosR version 1.12.0. LFQ intensities were filtered so that at least three valid values were present for either Kelly or KellyCis83 peptide samples. A two-step imputation process was utilised. First, imputation was performed on missing values within groups that had at least 50% availability. Group-specific imputation values were sampled from an empirical normal distribution within each group. For the remaining missing values, imputation was performed using small values derived from the lower tail of the sample distribution.

### 2.11. Bioinformatics and Network Analysis

Differentially abundant proteins for each data type (sEV, nuclear, and cytoplasm) were identified from normalised LFQ data with limma version 3.58.1 [26]. Differentially abundant proteins were defined based on the following criteria: adjusted *p* < 0.05 and absolute fold-change > 1.5. Gene set enrichment analyses were performed with clusterProfiler using Pathway Commons and Gene Ontology: Biological Process (GO: BP) databases [27,28,29]. A network of functionally related, significantly enriched pathways was created by inferring each pathway’s functional correlation using Jaccard distance with a minimum of 20% overlapping proteins using Enrich Plot version 1.22.0 [30].

### 2.12. Kaplan–Meier Survival Analysis

To assess the clinical significance of each shortlisted gene, the R2 Genomics Analysis and Visualisation Platform, which features built-in software, was utilised [31]. This is a web-based microarray data repository for tumour data submitted by various European institutes and developed by the Department of Human Genetics in the Amsterdam Medical Centre (AMC). The SEQC neuroblastoma tumour cohort comprises gene expression profiles from 498 primary neuroblastomas, obtained using both RNA-Seq (GSE49711) and microarrays (GSE49710) [32]. The SEQC available identifiers were sex, age at diagnosis, MYCN amplification status, risk status, INSS stage, disease course, tumour progression status, and death from disease.

This dataset was used for Kaplan–Meier analysis by gene expression, generating survival curves for both overall survival (OS), defined as the length of time from either the date of diagnosis or the start of treatment for a disease, indicating that patients diagnosed with the disease were still alive, and event-free survival (EFS), defined as the length of time after primary treatment for a cancer ends that the patient remains free of certain complications or events that the treatment was intended to prevent or delay. *p*-values were assessed for both survival outcomes, with a significance cut-off of *p* ≤ 0.05.

To assess the potential regulation of shortlisted genes by the MYCN oncogene, gene correlation analyses were run between MYCN and each gene using the SEQC cohort. Y-Y plots with Log2 transformation were generated with r-values and *p*-values.

### 2.13. NIR-AZA 1 Fluorophore sEV Labelling and Uptake

First, 2.0 × 10^4^ HUEVCs were seeded in a chambered coverslip (ibidi^®^, Cat #80826, Gräfelfing, Germany). After 24 h, we added KellyCis83’s sEVs, labelled with the near-infrared fluorophore NIR-AZA 1 as described previously [33]. The sEV-treated HUVECs were kept at 37 °C for 4 h. Then, the cells were washed twice with pre-warmed Hank’s Balanced Salt Solution (HBSS) and counterstained with 2 µg/mL of Hoechst in HBSS for 6 min. This was followed by two more washes with HBSS before confocal and fluorescent imaging. Images were processed using ImageJ (NIH, Maryland, MD, USA, version 1.53f51).

### 2.14. Lactate Secretion

The lactate secretion assay utilised the Lactate-Glo™ Assay (Promega, Cat #J5021, Madison, WI, USA) according to the manufacturer’s guidelines. Conditioned media were collected from Kelly, KellyCis83, and HUVECs treated with 10 μg of sEVs. Equal volumes of media and luciferin detection solution were mixed and incubated for 60 min at room temperature. Luminescence was measured on a PerkinElmer Victor3 plate reader (PerkinElmer, Hopkinton, MA, USA), with 5 s shaking and 0.2 s measurement time. Relative luminescence was measured by subtracting the luminescence of each test well from the blank, which consisted of non-conditioned culture media.

### 2.15. Seahorse XF Glycolytic Rate Assay

To measure the glycolytic rate of HUVECs treated with 10 μg of Kelly and KellyCis83 sEV, we performed the Seahorse XF Glycolytic Rate Assay (Agilent Technologies, Kit 103344-100), using the Seahorse XFe Analyser (Agilent Technologies, Chicopee, MA, USA), according to the manufacturer’s guidelines. HUVECs were plated at a density of 15 × 10^3^ cells per day prior to treatment with either Kelly or KellyCis83 sEVs. HUVECs and sEVs were co-cultured for 24 h before washing three times with assay medium containing DMEM, glucose, pyruvate, and L-glutamine. Next, 0.5 μM Rot/AA and 50 mM 2-DG were resuspended in the assay medium and loaded onto the sensor cartridge. Lastly, the cell plate and sensor cartridge were loaded onto the Seahorse XFe Analyser, and the Seahorse XF Glycolytic Rate Assay was performed.

### 2.16. Tubule Formation Assay

The tubule formation assay was performed by treating Human Umbilical Vein Endothelial Cells (HUVECs) cultured on Matrigel^®^ Growth Factor Reduced Basement Membrane Matrix (Corning^®^ Cat #354230, Tewksbury, MA, USA) with 20 μg of Kelly and KellyCis83 sEVs. To preserve the differentiating properties of HUVECs, cells were kept in a passage range of 2–8 for all downstream analysis. To create positive and negative culture conditions for the functional analysis of HUVECs, culture media conditions were designed (Table 1).

Each well was imaged in two locations, every hour, for 24 h on a ZEISS CellDiscoverer 7 microscope (Carl Zeiss Ltd., Cambridge, UK). Tubule formation was measured stereoscopically using ImageJ (NIH, MD, USA, version 1.53f51). The number of times a HUVEC tubule or part of the cell body fully crossed an overlayed grid of 114,344.90 µm^2^ was counted, and tubule formation was reported as the number of HUVEC tubule grid intersections.

### 2.17. Wound Healing Assay

A reproducible 500 μm cell-free scratch in a monolayer of HUVECs was created using silicone 2-well inserts (ibidi^®^, Cat #80209, Gräfelfing, Germany), placed in a 48-well plate. Into each well of each insert, 5 × 10^4^ HUVECs were seeded. At 48 h post-seeding, the inserts were removed, cells were washed with pre-warmed PBS and treated with 20 μg of sEVs. Each scratch was imaged in three locations every hour for 24 h on a ZEISS CellDiscoverer 7 microscope (Carl Zeiss Ltd., Cambridge, UK). The scratch area was measured using the area measurement tool on ImageJ (NIH, MD, USA, version 1.53f51). The following formula was used to determine the percentage of scratch or wound closure:

$$Wound\;Closure\;=\;\frac{\left(Scratch\;area\;at\;0\;h\;-\;Scratch\;area\;at\;10\;h\right)}{-Scratch\;area\;at\;0\;h\;}\cdot 100\%$$

### 2.18. Cell Proliferation

Cell proliferation was assessed using the Quant-iT PicoGreen dsDNA Assay Kit (Invitrogen, Cat #P7589, Carlsbad, CA, USA) as per the manufacturer’s instructions. Into a 96-well plate, 1.0 × 10^4^ cells were seeded and treated with sEVs at 24 and 48 h after plating. On day five, after the last sEV treatment, the media was removed; the cells were washed with pre-warmed PBS and then lysed with dsDNA extraction buffer (0.1 M sodium bicarbonate and 1% Triton-X, Cat A16046, Ward Hill, MA, USA) for 30 min before collection. Each dsDNA sample was diluted 1:4 in TE buffer (200 mM Tris-HCl, 20 mM EDTA, pH 7.5), and diluted PicoGreen™ reagent (1:200 in TE buffer, Cat 12090015, Carlsbad, CA, USA) was added. The results were measured using the Tecan i-control infinite 200Pro plate reader (Tecan Group Ltd., Männedorf, Switzerland).

### 2.19. Half Maximal Inhibitory Concentration (IC_50_) Determination

To determine the half maximal toxic concentration (IC_50_) of cisplatin, we plated 1.0 × 10^4^ Kelly, KellyCis83, and SH-SY5Y cells in 96-well plates. After 24 h, cells were washed with PBS and treated with five concentrations of cisplatin, ranging from 0.5 µM to 10 µM, including an untreated control. Cell viability was determined by using the CellTiter-Glo^®^ 3D Cell Viability Assay (Promega, Cat #G9681, Madison, WI, USA) to measure cellular ATP levels and percentage of viability relative to the untreated control. The IC_50_ was calculated on GraphPad Prism (GraphPad Software, Solana Beach, CA, USA, version 9.0.0) by non-linear regression (curve fit) with a baseline set to 0, plotting percentage viability on the y-axis and log10 cisplatin concentration on the x-axis.

### 2.20. Cisplatin Toxicity Assay

We assessed the ability of Kelly and KellyCis83 sEVs to inhibit cisplatin toxicity in Kelly and SH-SY5Y cells. We plated 1.0 × 10^4^ Kelly and SH-SY5Y cells in 96-well plates and treated them twice with 8 µg of Kelly and KellyCis83 sEVs. After 24 h, the cells were treated with 2 µM of cisplatin. After 5 days, the percentage of cell viability was measured relative to the untreated control using the CellTiter-Glo^®^ 3D Cell Viability Assay (Promega, Cat #G9681, Madison, WI, USA).

### 2.21. Statistics

Data were analysed using GraphPad Prism 10 (GraphPad Software, USA). To compare (i) the means of two groups, an unpaired two-tailed *t*-test with the Mann–Whitney correction was used, and (ii) the means of multiple groups, an ordinary one-way ANOVA was used, with a *p* value indicating statistical significance. These tests were performed on all data showing statistical significance, as highlighted, unless noted otherwise in the figure legends. Graphed *p* values are represented as follows: * = *p* ≤ 0.05, ** = *p* ≤ 0.01, *** = *p* ≤ 0.001 and **** = *p* ≤ 0.0001.

## 3. Results

### 3.1. Purification and Physical Characterisation of sEVs Derived from Kelly and KellyCis83 NB Cells

**Figure 1:**
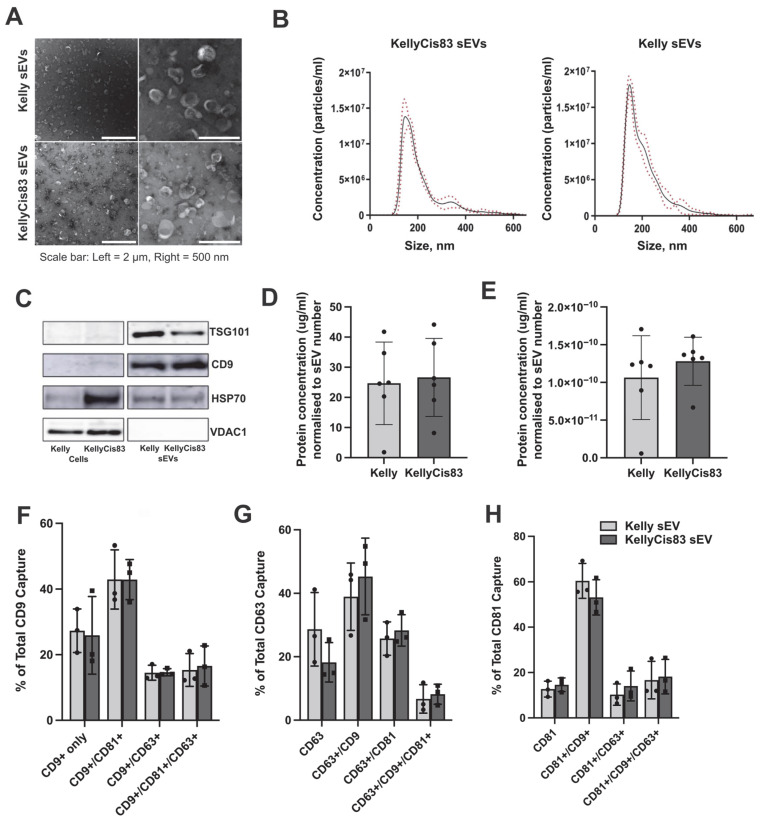
Kelly and KellyCis83 sEV characterisation: (**A**) TEM images of Kelly sEV and KellyCis83 sEV (scale bar = 2 μm and 500 nm); (**B**) NTA size range of Kelly sEV and KellyCis83 sEVs (*n* = 3, N represents biological replicates of sEVs). Data are represented as mean ± standard deviation (red dotted line); (**C**) representative blot of three independent experiments, showing expression of CD9, HSP70, TSG101, and VDAC-1 in Kelly and KellyCis83 parental cells and sEVs. Images for VDAC-1 and TSG101 are derived from the same blot, cut into two, prior to primary antibody incubation. The same is for CD9 and HSP70 images. (**D**) Number of sEVs produced by Kelly and KellyCis83 cells, normalised to cell number; (**E**) protein concentration of Kelly and KellyCis83 sEVs, normalised to sEV number; (**F**) co-localisation of CD9, CD81, and CD63 on Kelly and KellyCis83 sEVs captured by anti-CD9, (**G**) anti-CD63, and (**H**) anti-CD81 antibodies on the ExoView platform. Data are represented as mean ± standard deviation. Statistical significance was calculated by an unpaired *t*-test and an ordinary one-way ANOVA. No statistically significant results were detected.

Kelly and KellyCis83 sEVs cultured in CELLine AD1000 bioreactors were isolated by differential ultracentrifugation and characterised by nanoparticle tracking analysis (NTA), transmission electron microscopy (TEM), and Western blot (WB), fulfilling the MISEV 2023 guidelines for characterisation of small extracellular vesicles [20]. We observed spherical particles with a bright outer ring and a darker central area using TEM (Figure 1A). The diameters of the sEV samples ranged from 132 to 175 nm. Kelly’s sEVs had a mean diameter of 206 ± 3 nm and a mode diameter of 147 ± 3 nm. KellyCis83 sEV had a mean diameter of 216 ± 4 nm and a mode diameter of 155 ± 7 nm (Figure 1B). Western blot results showed that both Kelly and KellyCis83 sEVs are enriched in CD9 and TSG101, compared to their parental cells (Figure 1C). HSP70 was expressed in sEVs, but its level of enrichment was not as high as that of the parental cells. VDAC-1, a mitochondrial membrane-bound protein, was not detected in Kelly or KellyCis83 sEVs, showing that the sEV isolates were free from cellular contamination. Comparison of the number and protein concentration of sEVs isolated from Kelly and KellyCis83 cells revealed no significant differences when normalised to the number of cells at conditioned media harvest (Figure 1D,E).

Using the ExoView R200 platform, we examined the surface topology of single Kelly and KellyCis83 sEVs. We performed a tetraspanin antibody array, detecting expression and co-localisation of CD9, CD81, and CD63 (Figure 1F–H). CD9 is the dominant tetraspanin expressed on the surface of both Kelly and KellyCis83 sEVs (Figure 1F). The CD9+/CD81+ phenotype is the most common tetraspanin phenotype in both types of sEVs. Triple tetraspanin-positive sEVs account for only 3–27% of sEVs (Figure 1G,H). These results show that while the development of cisplatin resistance profoundly reshaped the genome and proteome of the KellyCis83 cell line [17], it did not significantly alter sEV tetraspanin expression.

### 3.2. Functional Enrichment Analysis of sEV Proteome Identifies Metabolism as a Key Dysregulated Pathway in KellyCis83 sEVs

**Figure 2:**
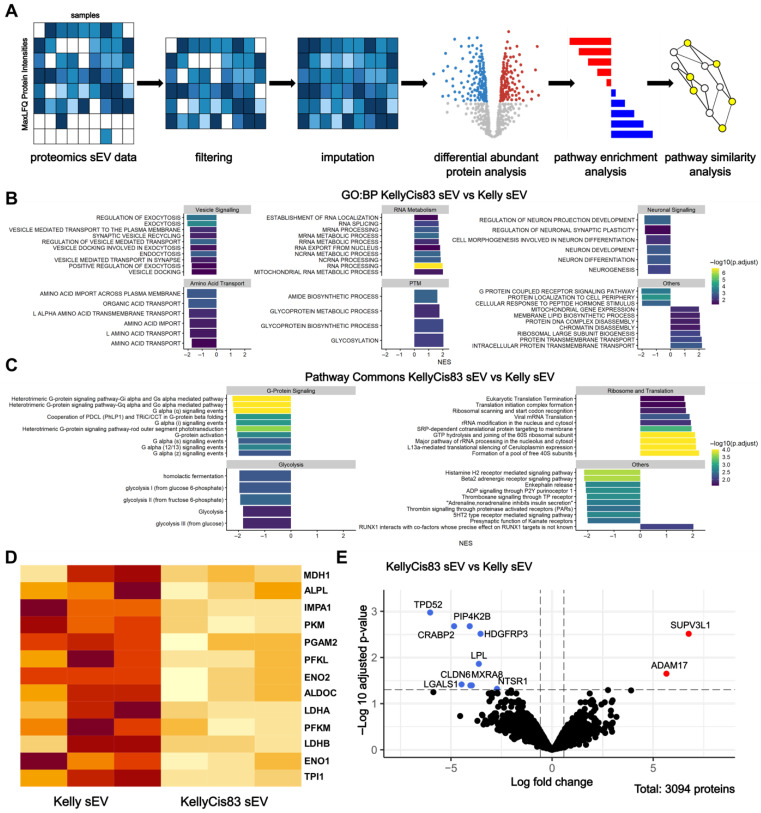
Mass spectrometry and bioinformatic analysis of Kelly and KellyCis83 sEVs: (**A**) bioinformatics workflow for sEV proteomics analysis in Kelly and KellyCis83 cells; (**B**) gene set enrichment analysis using the Gene Ontology: Biological Processes (GO: BP) and (**C**) Pathway Commons databases. The colour gradient indicates the –log10-adjusted *p* value, and the x-axis indicates the normalised enrichment score (NES). Significant pathways (*p* < 0.05) are grouped based on the higher-level classifications, and the top 10 most significant are visualised. (**D**) Heatmap of normalised LFQ intensities of proteins that contributed to significant glycolysis enrichment. A darker orange colour indicates higher protein abundance. Individual proteins were not differentially abundant in KellyCis83 sEVs; (**E**) volcano plot displaying differential abundance of KellyCis83 and Kelly sEV proteins (adjusted *p* < 0.05, absolute fold change > 1.5).

To further characterise Kelly and KellyCis83 sEV content and determine protein expression changes as a result of cisplatin resistance development, we performed liquid chromatography with tandem mass spectrometry (LC-MS/MS) on Kelly and KellyCis83 sEVs, followed by bioinformatic analysis using clusterProfiler (Figure 2A). Data were filtered and imputed. This resulted in the identification of a total of 3094 proteins (Figure 2E). To gain insight into the different biological and molecular functions of proteins differentially expressed in our cisplatin-resistant model of NB, we performed Gene Set Enrichment Analysis (GSEA) followed by pathways similarity analysis and network visualisation (Figure 2B,C). Differentially abundant proteins between Kelly and KellyCis83 sEVs were denoted with an absolute fold change of >1.5 and an adjusted *p* value of <0.05. With this threshold, we identified nine downregulated proteins (TPD52, PIP4K2B, CRABP2, HDGFRP3, LPL, MXRA8, CLDN6, LGALS1, and NTSR1) and two upregulated proteins (SUPV3L1 and ADAM17) in KellyCis83 sEVs (Figure 2E, Supplementary Figure S1, Supplementary Table S2).

We identified a group of pathways that were significantly downregulated in KellyCis83 sEVs related to vesicle signalling, amino acid transport, neuronal signalling, and glycolysis. Conversely, we identified RNA metabolism and post-translational modification to be significantly upregulated in KellyCis83 sEVs (Figure 2B,C). We further investigated the downregulation of glycolysis in KellyCis83 sEVs, a sign of metabolic dysregulation. Although glycolysis proteins were not found to be differentially abundant in KellyCis83 sEVs, we identified several proteins contributing to the Gene Set Enrichment Analysis of the significantly downregulated glycolysis pathway, such as ENO1, ENO2, ALDOC, LDHA, and LDHB (Figure 2D).

### 3.3. KellyCis83 sEVs Are Internalised by Cancerous and Non-Cancerous Cells

**Figure 3:**
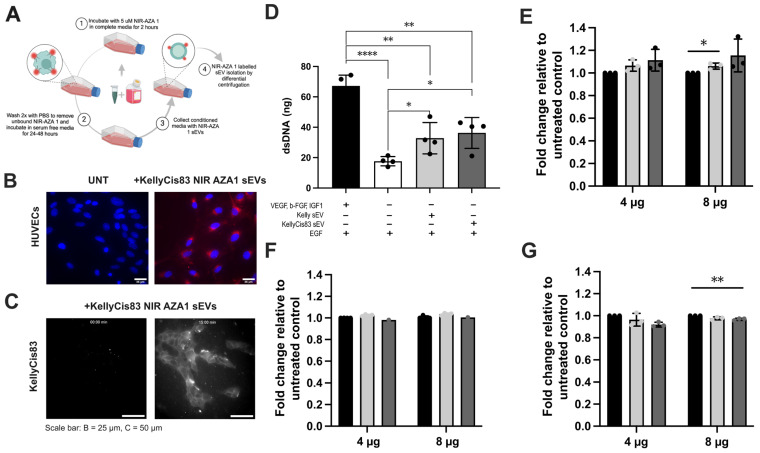
sEVs taken up by cancerous and non-cancerous cells affect cell proliferation. (**A**) Schematic representation of NIR-AZA 1 labelling protocol; image created with Biorender.com. (**B**) Confocal imaging of HUVECs fixed in PFA and counterstained with Hoechst after incubation with PBS (untreated) or KellyCis83 NIR-AZA 1 sEVs. Scale bar = 25 µm; (**C**) live fluorescent microscopy imaging time lapse of KellyCis83 NIR-AZA 1 sEV uptake in unlabelled KellyCis83 cells at 0 min and 15 min. Scale bar = 50 µm; (**D**) proliferation of HUVECs, measured by dsDNA, 5 days after treatment with 4 μg Kelly’s sEVs and KellyCis83’s sEVs (*n* = 3, N represents biological replicates). The bar colour coding for treatments: positive control (GF+) is black, negative control (GF−) is white, Kelly sEV treatment is light grey, and KellyCis83 sEV treatment is dark grey. (**E**) Fold change in cell proliferation (dsDNA) of SH-SY5Y, (**F**) Kelly, and (**G**) KellyCis83 cells five days after treatment with 4 μg or 8 μg of Kelly and KellyCis83 sEVs. Data collected from three technical replicates. Bar colour coding for treatments: untreated is black, Kelly’s sEV is light grey, and KellyCis83’s sEV is dark grey. Data are represented as mean ± standard deviation of fold change relative to untreated control. Statistical significance was calculated by an ordinary one-way ANOVA (* = *p* ≤ 0.05, ** = *p* ≤ 0.01, **** = *p* ≤ 0.0001, only statistically significant values graphed).

To visualise sEV uptake by cancerous (KellyCis83 cells) and non-cancerous (human umbilical vein endothelial cell (HUVEC)) recipients, we produced near-infrared fluorescent KellyCis83 sEVs using the previously reported endogenous labelling method with the NIR-AZA 1 fluorophore [33] (Figure 3A). Next, we incubated HUVECs with either PBS or NIR-AZA 1-labelled KellyCis83 sEVs in 5% CO_2_ at 37 °C. After 3 h, the HUVECs were washed with HBSS, fixed in paraformaldehyde (PFA), counterstained with Hoechst, and then visualised using confocal microscopy (Figure 3B). Cellular fluorescence of recipient KellyCis83 cells was observed following 15 min of incubation with NIR-AZA 1-labelled KellyCis83 sEVs. Cells were imaged by live widefield fluorescent microscopy at 0 min and 15 min (Figure 3C).

### 3.4. Cell Proliferation Demonstrated a Dose-Dependent Response to sEV Treatment

We then investigated whether the active uptake of Kelly and KellyCis83 sEVs in cancerous and non-cancerous cells affects their proliferation. We observed increased proliferation in HUVECs treated with Kelly and KellyCis83 sEVs (Figure 3D). HUVECs cultured in HUVEC media lacking the angiogenic factors VEGF, IGF-1, and b-FGF (GF−) were treated with 4 μg Kelly and KellyCis83 sEVs 24 h post-seeding. HUVECs cultured in GF+ and GF− media with an equal volume of PBS in place of treatment were used as positive and negative controls. After five days of culture, dsDNA was measured to ascertain the HUVEC rate of proliferation. The proliferative rate of HUVECs treated with Kelly and KellyCis83 sEVs was increased by >16% (*p* = 0.04) and >30% (*p* = 0.03), respectively, compared to the untreated negative control.

NB cells with a different *MYCN* status were then treated with 4 μg and 8 μg of Kelly and KellyCis83 sEVs (Figure 3E–G). When treating *MYCN*-amplified Kelly and KellyCis83 cells with sEVs, Kelly cells had no significant change in proliferation. However, the proliferation rate of KellyCis83 cells decreased by 8% (*p* = 0.007) and 3.2% (*p* = 0.0092) when treated with 4 μg and 8 μg of KellyCis83 sEVs, respectively, compared to the untreated control. Conversely, non-*MYCN*-amplified SH-SY5Y cells had an observable increase in proliferation compared to the untreated control, an effect that was enhanced with higher quantities of KellyCis83 sEVs. The proliferation of SH-SY5Y cells increased by 14% (*p* = 0.005) and 18% (*p* = 0.002) with Kelly and KellyCis83 sEV treatment, respectively. The characteristic of Kelly and KellyCis83 sEVs with amplified *MYCN*, a known oncogenic transcription factor, may be responsible for this effect in non-*MYCN*-amplified cells.

### 3.5. sEVs Secreted by NB Cells Increased Lactate Secretion and Altered Metabolic Pathways in HUVECs

**Figure 4:**
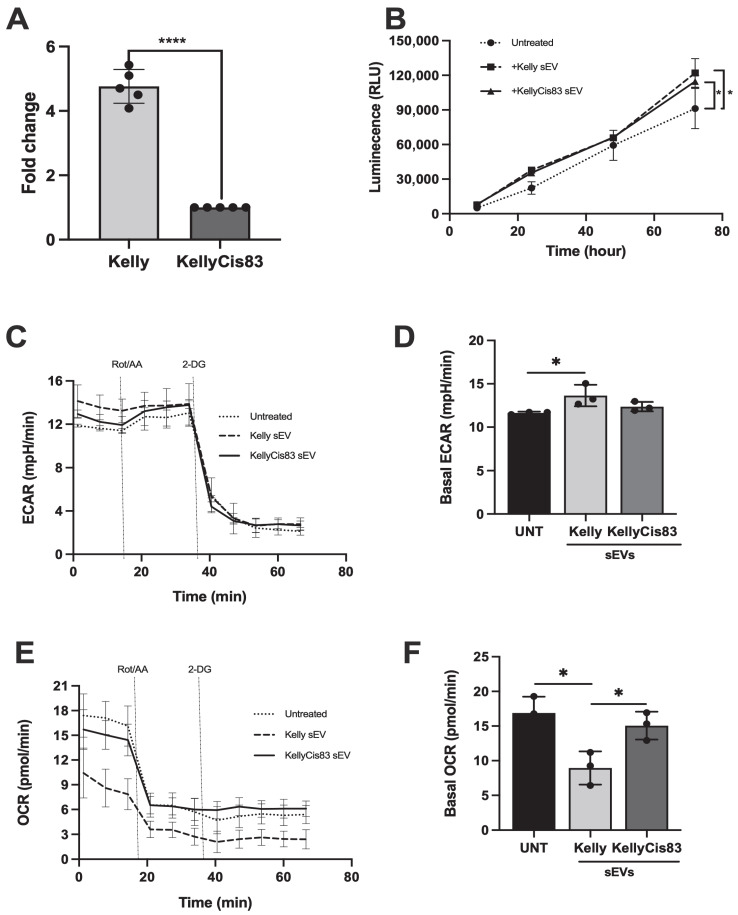
Metabolic reprogramming of HUVECs by Kelly and KellyCis83 sEVs. (**A**) Lactate secretion in Kelly and KellyCis83 cells is represented as fold change relative to KellyCis83 after normalisation to dsDNA yield. Data are represented as the mean ± standard deviation of 5 technical replicates; (**B**) lactate secretion of HUVECs measured at 8, 24, 48, and 72 h after treatment with 10 μg of Kelly and KellyCis83 sEVs. (**C**,**D**) Seahorse glycolytic rate assay: extracellular acidification rate and (**E**,**F**) oxygen consumption rate of HUVECs treated with 10 µg of Kelly and KellyCis83 sEVs (*n* = 3, N represents biological replicates). The bar colour coding for treatments: untreated is black, Kelly’s sEVs is light grey, and KellyCis83’s sEVs is dark grey. Data are represented as the mean ± standard deviation. Statistical significance was calculated by a Student’s unpaired *t*-test and an ordinary one-way ANOVA (* = *p* ≤ 0.05 and **** = *p* ≤ 0.0001, only statistically significant values graphed).

Proteomic analysis of Kelly and KellyCis83 cells and their corresponding sEVs identified several dysregulated biological pathways related to metabolism and glycolysis (Figure 2). To validate this altered metabolic pathway, we analysed lactate secretion, a metabolite of the glycolysis pathway closely related to cell proliferation, in Kelly and KellyCis83 cells. To control for changes in proliferation due to culture media and sEV treatment, we normalised lactate secretion to dsDNA yield. After 72 h of culture, Kelly cells secreted >4.7 fold (*p =* 0.0001) more lactate compared to KellyCis83 (Figure 4A). To determine if sEVs could alter lactate secretion in cells of the NB microenvironment, we treated HUVECs with 10 μg of Kelly and KellyCis83 sEVs. Lactate secreted from HUVECs was measured at 8, 24, 48, and 72 h. Lactate secretion in HUVECs 72 h post sEV treatment increased by 33% (*p* = 0.03) and 25% (*p* = 0.04) when treated with Kelly and KellyCis83 sEVs, respectively, compared to the untreated negative control (Figure 4B). This indicates that Kelly and KellyCis83’s sEVs can metabolically reprogram endothelial cells. To confirm this finding further, we performed the Seahorse XF Glycolytic Rate Assay on HUVECs treated with 10 µg of Kelly and KellyCis83 sEVs 48 h prior to analysis on the Seahorse XF Analyser. We found that Kelly’s sEVs significantly increased the extracellular acidification rate (ECAR) of HUVECs compared to the untreated control (Figure 4C,D). While the EACR of HUVECs treated with KellyCis83 sEVs increased compared to the untreated control, no difference was observed compared with Kelly sEV-treated HUVECs. Conversely, Kelly’s sEVs significantly decreased the oxygen consumption rate (OCR) of HUVECs, but KellyCis83’s sEVs compared to the untreated control (Figure 4E,F). This demonstrates that Kelly’s sEVs increased the glycolytic rate and decreased the oxidative phosphorylation rate in HUVECs, potentially due to the enrichment of sEVs with glycolytic enzymes (Figure 2).

### 3.6. KellyCis83 sEVs Increased Anchorage-Dependent Differentiation of HUVECs

**Figure 5:**
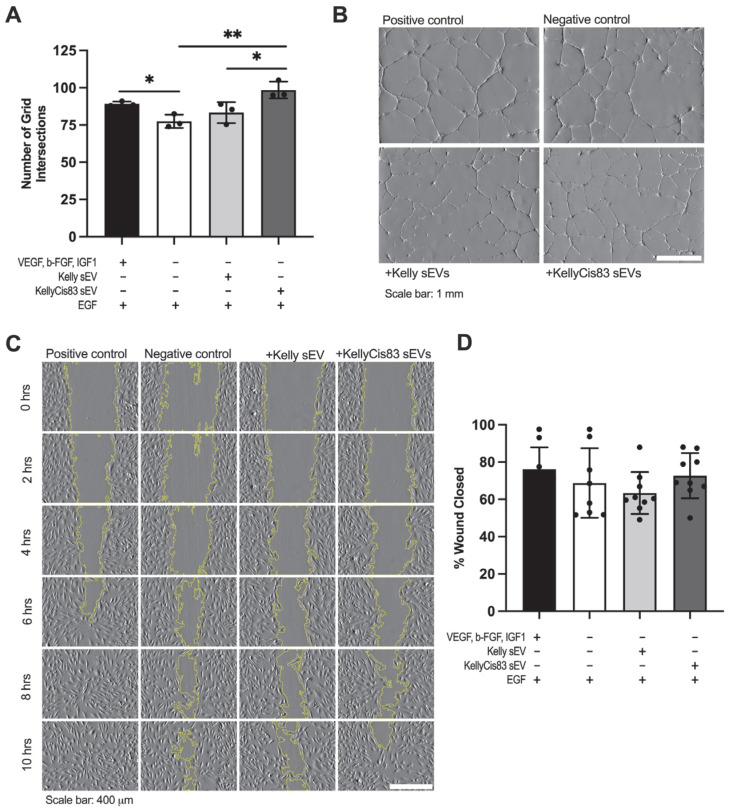
Anchorage-dependent differentiation of HUVECs treated with Kelly and KellyCis83 sEVs. (**A**) Tubule formation in HUVECs 24 h after treatment with 20 μg of Kelly and KellyCis83 sEVs measured by the number of tubule intersections with a 114,344.90 μm^2^ grid. Graphs display the mean ± standard deviation of three technical replicates; (**B**) representative images of tubule formation in HUVECs in GF+ and GF− media treated with PBS and HUVECs in GF− media treated with Kelly’s sEVs and KellyCis83 sEVs. Scale bar = 1 mm; (**C**) representative images of HUVECs in scratch assay with Kelly and KellyCis83 sEV treatment over 10 h. Scale bar = 400 μm; (**D**) percentage of wound closure 10 h after treatment of HUVECs with 8 μg Kelly and KellyCis83 sEVs. Graphs display the mean ± standard deviation of three technical replicates. The bar colour coding for treatments: positive control (GF+) is black, negative control (GF−) is white, Kelly’s sEV treatment is light grey, and KellyCis83’s sEV treatment is dark grey. Statistical significance was calculated by a Student’s unpaired *t*-test and an ordinary one-way ANOVA (* = *p* ≤ 0.05 and ** = *p* ≤ 0.01, only statistically significant values graphed).

After confirming the ability of NB sEVs to alter cell proliferation and metabolism of HUVECs, we decided to test the ability of Kelly and KellyCis83 sEVs to trigger their anchorage-dependent differentiation. We performed a tubule formation assay by seeding HUVECs on Matrigel in HUVEC media lacking the angiogenic factors VEGF, b-FGF, and IGF-1 (GF−). The cells were then treated with 20 μg of Kelly or KellyCis83 sEVs. The negative control (GF−) and positive control (GF+) were treated with an equal volume of PBS. After 24 h, we performed a stereoscopic analysis and counted each instance in which a tubule intersected a 114,344.90 μm^2^ grid. We demonstrated that GF− media significantly decreased tubule formation compared to HUVECs cultured in GF+ media containing angiogenic factors (Figure 5A). We identified amplified tubule formation in HUVECs cultured in GF− media in response to KellyCis83 sEV treatment. HUVECs treated with KellyCis83 had 27% more tubule formation (*p* = 0.007) compared to the negative control and 18% more compared to HUVECs treated with Kelly’s sEVs (*p* = 0.04) (Figure 5A,B).

To verify that the observed anchorage-dependent differentiation abolishes HUVECs’ migration in response to Kelly and KellyCis83 sEV exposure, we performed a scratch assay. We measured the percentage of wound closure after 10 h. In GF+ media, 25,000 HUVECs were seeded into each well of a 0.22 cm^2^ insert. After 24 h, the insert was removed, and cells were treated with 8 μg of Kelly or KellyCis83 sEVs and GF− media. The positive and negative control media were replaced with GF+ and GF− media, respectively. While no significant changes in migration were observed when HUVECs were exposed to Kelly or KellyCis83 sEVs compared to the negative control, KellyCis83 sEVs had an observed higher capacity of migration in comparison to Kelly sEVs by 10 h (Figure 5C,D). Either an increased amount of KellyCis83 sEVs or more frequent treatment with sEVs may further stimulate cell migration.

### 3.7. Transfer of Chemoresistance via KellyCis83 sEVs

To determine the ability of sEVs derived from KellyCis83 cells to transfer the cisplatin resistance phenotype to cisplatin-sensitive NB Kelly and SH-SY5Y cells, we treated Kelly and SH-SY5Y cells with Kelly and KellyCis83 sEVs in conjunction with cisplatin.

**Figure 6:**
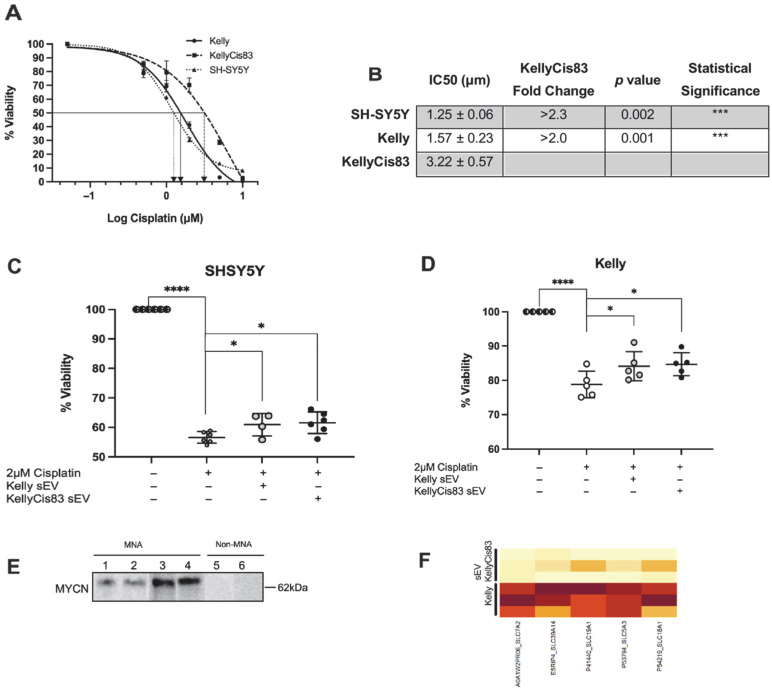
Kelly and KellyCis83 sEVs can reduce cisplatin toxicity in NB cells. (**A**) Cisplatin dose response curve in Kelly, KellyCis83, and SH-SY5Y cells. Data are represented as mean ± standard deviation of three technical replicates; (**B**) IC_50_ values of Kelly, KellyCis83, and SH-SY5Y cells treated with cisplatin. IC_50_ fold change values are relative to KellyCis83 IC_50_; (**C**) percentage viability of SH-SY5Y and (**D**) Kelly cells treated with two doses of 8 µg Kelly and KellyCis83 sEVs and 2 µM cisplatin. Viability was measured by cellular ATP production 5 days post-cisplatin treatment. Data are represented as mean ± standard deviation of a minimum of four technical replicates. Circle colour coding for treatments: untreated is half black, cisplatin treated is white, Kelly’s sEVs is light grey, and KellyCis83’s sEVs is dark grey. (**E**) Western blot results of NB cell line-derived sEVs examined with MYCN target. MYCN-amplified NB-derived sEVs: (1) KellyCis83, (2) Kelly, (3) SK-N-BE(2), and (4) NB-1691. Non-MYCN-amplified NB-derived sEVs: (5) SH-SY5Y and (6) SK-N-AS. Images for MYCN-enriched sEVs are derived from different blots but incubated with the primary antibody in the same tray. (**F**) Bioinformatic analysis of Kelly and KellyCis83 sEV proteome for altered drug transporters. A darker orange colour indicates a higher protein abundance. Individual proteins were not differentially abundant in KellyCis83 sEVs. Statistical significance was calculated by a Student’s unpaired *t*-test and an ordinary one-way ANOVA (* = *p* ≤ 0.05, *** = *p* ≤ 0.001 and **** = *p* ≤ 0.0001, only statistically significant values graphed).

The appropriate dose of cisplatin for this experiment was extracted from the cisplatin dose response curve for Kelly, KellyCis83, and SH-SY5Y (Figure 6A,B). The half-maximal inhibitory concentrations (IC_50_) were determined by fitting a non-linear regression curve, with a baseline of zero, to the percentage of viable cells after increasing doses of cisplatin. The IC_50_ values of cisplatin treatment were 3.22 µM for KellyCis83 cells, 1.57 µM for Kelly cells, and 1.25 µM for SH-SY5Y cells. When compared to Kelly and SH-SY5Y cells, the KellyCis83 IC_50_ was > 2-fold higher (*p* = 0.001) and >2.3-fold higher (*p* = 0.002), respectively.

Kelly and SH-SY5Y cells were treated twice with 8 µg sEVs (total of 16 µg sEV treatment), in the 48 h prior to treatment with 2 µM of cisplatin, and their approximate IC_50_ concentrations of cisplatin. Five days post cisplatin treatment, we measured cellular adenosine triphosphate (ATP) to calculate cellular viability. There was an average increase of 4.3% *(p* = 0.04) and 5.0% (*p* = 0.01) in SH-SY5Y viability with Kelly and KellyCis83 sEV treatment, respectively (Figure 6C). We observed a 6.08% (*p* = 0.04) increase in the viability of Kelly cells treated with Kelly sEVs and an increase of 6.62% (*p* = 0.02) with KellyCis83 sEV treatment (Figure 6D). Although the increase in cell viability is statistically significant, its biological effect is relatively small and warrants further investigation.

The sEVs isolated from Kelly and KellyCis83 cells were found to similarly protect NB cells from the effects of cisplatin, possibly due to the amplification of the *MYCN* oncogenic transcription factor. Western blot analysis of sEVs derived from NB cells confirmed *MYCN*-enrichment in sEVs isolated from *MYCN* amplified NB cell lines, such as KellyCis83, Kelly, SK-N-BE(2), and NB-1691 sEVs, in contrast to the non-*MYCN*-amplified SH-SY5Y and SK-N-AS sEVs (Figure 6E, Supplementary Figure S2, Supplementary Table S3). While bioinformatic analysis did not find any significant upregulation of drug transporters in KellyCis83 sEVs, it found several solute carrier (SLC) proteins that contribute to significantly enriched pathways, mainly related to amino acid transport, suggesting dysregulated cellular transport in KellyCis83 sEVs (Figure 6F). The lack of significant changes in drug transporter expression suggests that sEV protective effects are not due to altered cisplatin transport.

### 3.8. Clinical Relevance of Proteins That Contributed to Significant Glycolysis Enrichment in Neuroblastoma

The molecular components of sEVs, especially exosomal proteins, are promising candidates as circulating biomarkers for the clinical diagnosis and prognosis of cancer and tumour response to therapy [34,35,36]. Here, we evaluated the clinical relevance of the identified glycolytic proteins in Kelly’s and KellyCis83’s sEVs (Figure 2D) using the R2: Genomics Analysis and Visualisation Platform [31]. We correlated the mRNA expression of selected genes with Event-Free Survival (EFS) and Overall Survival (OS) in the SEQC cohort of 498 neuroblastoma cases, applying Kaplan–Meier analysis (Table 2) [32]. Out of 13 targets, the high expression of 9 (*ALPL*, *ENO1*, *IMPA1*, *LDHA*, *LDHB*, *MDH1*, *PFKM*, *PKM*, *TPI1*) was strongly associated with poor EFS and OS, although *IMPA1* was not associated with OS. The low expression of *ALDOC*, *ENO2*, *PGAM2*, and *PFKL* was strongly associated with poor EFS and OS.

About 20% of neuroblastomas exhibit amplification of the *MYCN* oncogene, which is the most well-characterised oncogenic driver and a key indicator of poor prognosis in neuroblastoma, important for risk group stratification in this disease [37,38,39]. Consequently, we also correlated the expression of the shortlisted glycolytic candidates with *MYCN* mRNA in the SEQC cohort of 498 neuroblastomas. While *LDHB* showed the strongest positive correlation with *MYCN* expression (r-value = 0.708, *p* = 4.73 × 10^−77^), no correlation was observed for ALPL. The positive correlation of *ENO1*, *LDHA*, *LDHB*, *PFKM*, *PKM*, and *TPI1* with *MYCN* may suggest that their expression can be driven by MYCN and their subsequent co-localisation in sEVs, which may not be the case for *ALDOC*, *ENO2*, *IMPA*, and *PGAM2*, which have an inverse correlation with *MYCN* expression.

**Figure 7:**
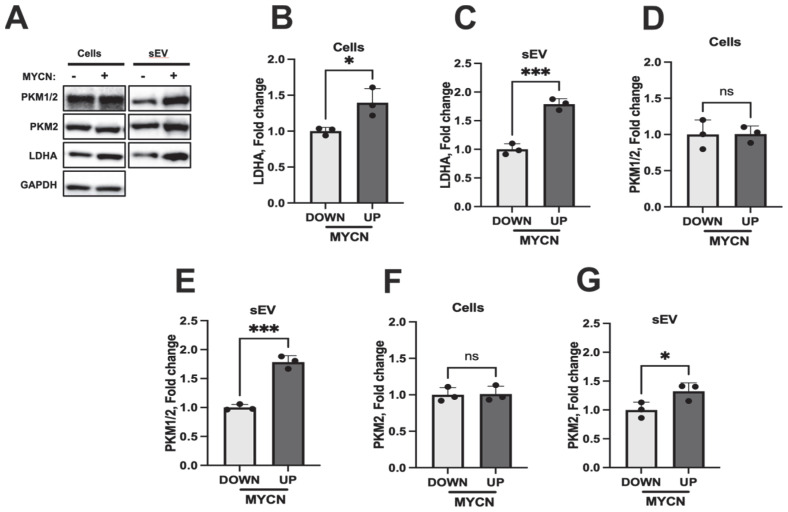
MYCN-inducible co-expression of LDHA, PKM1/2 and PKM2. (**A**) Representative blot of three independent experiments, showing expression of PKM1/2, PKM2, and LDHA in MYCN-UP/DOWN parental cells and corresponding sEVs. Images are derived from the different blots, prior to primary antibody incubation. Densitometry analysis of LDHA ((**B**) cellular lysates and (**C**) sEVs), PKM1/2 ((**D**) cellular lysates and (**E**) sEVs), and PKM2 ((**F**) cellular lysates and (**G**) sEVs) detection. Data are represented as mean ± standard deviation. Statistical significance was calculated by a Student’s unpaired *t*-test (* *p* ≤ 0.05 and *** *p* ≤ 0.001, only statistically significant values graphed; ns: not significant).

Having identified *MYCN* positively correlated genes, we then tested some of them, e.g., *PMKs* and *LDHA*, investigating whether they were co-localised in sEV using the doxycycline-inducible neuroblastoma cell line [22]. We probed cell lysates and sEVs from SHEP-Tet21N cells grown both in the presence and absence of doxycycline for LDHA using a Western blot. The addition of doxycycline resulted in ~5-fold lower expression of MYCN in SHEP-Tet21N cells (*p* < 0.05), confirming oncogene repression (Supplementary Figure S3). We experimentally validated the MYCN-driven expression of cellular LDHA, PMK2 and PMK1/2 in vitro (Figure 7). While a marked increase in cellular LDHA expression by ~40% (*p* < 0.05) was detected, no difference was observed for cellular PKM1/2 and PKM2. Intriguingly, we found that LDHA (by 1.8-fold change, *p* < 0.001), PKM2 (by 1.2-fold change, *p* < 0.05), PKM1/2 (by 1.8-fold change, *p* < 0.001), and MYCN (by 4-fold change, *p* < 0.05) were co-localised in sEV fractions of SHEP-Tet-21N cells overexpressing MYCN (Figure 7A, Supplemental Figure S3). Interestingly, MYCN-UP sEVs showed stronger enrichment for LDHA, PKM1/2, and PKM2 compared to the corresponding cells, suggesting a MYCN-driven mechanism of sEV enrichment.

## 4. Discussion

Very little research has been conducted on how chemoresistant cancer cells influence sEV signalling in NB. In this study, we employed an in vitro model of a cisplatin-resistant NB (KellyCis83) developed in our lab [17,18,19] to characterise changes in the sEV proteome, to evaluate associated phenotypic changes and to investigate the potential for chemoresistance transfer promoted by sEVs. The number of differentially expressed proteins in KellyCis83 sEVs suggests functional shifts that could alter communication with recipient cells. Our findings are supported by a recent study demonstrating an altered exosomal protein composition in response to ionising radiation of NB SH-SY5Y cells [40]. An increase in viability, migration, and radio resistance in non-irradiated SH-SY5Y cells treated with irradiation-induced EVs was reported. This suggests that sEVs secreted by cisplatin-resistant NB cells may promote similar pro-survival or pro-tumourigenic signals in various recipient cell types.

In KellyCis83’s sEVs, the majority of altered pathways were related to glycolysis, mirroring the pathways identified in the parental cells in the early work by Piskareva et al. Ingenuity Pathway Analysis, which studied the identified enrichment of proteins in KellyCis83 cells related to sucrose degradation, glycolysis, and gluconeogenesis [17]. Further validation of proteomics and gene ontology results demonstrated that cisplatin-sensitive Kelly cells produced >4.7 fold more lactate than cisplatin-resistant KellyCis83 cells, indicating a metabolic shift. The cisplatin-resistant modulation of the glycolysis effect has also been reported in ovarian cancer [41]. Growing evidence suggests that tumour cells undergo metabolic reprogramming to survive and promote resistance to chemotherapy agents [42,43,44]. The metabolic symbiosis phenomenon describes how tumour cells switch between aerobic glycolysis and OXPHOS, depending on the tumour microenvironment (TME) and amplified oncogenes such as *MYCN* [44,45]. Both increased glycolysis and reduced OXPHOS are metabolic features characteristic of hypoxic cancer cells [43], which were more pronounced in Kelly’s sEVs compared to KellyCis83’s sEVs. Interestingly, both Kelly and KellyCis83 sEVs reprogrammed HUVECs to increase lactate secretion at equivalent levels. While the sEV-induced aerobic glycolytic rate was similar, oxidative phosphorylation (OXPHOS) was significantly reduced in HUVECs treated with Kelly’s sEVs compared to KellyCis83’s sEVs, which may be due to an altered balance of glycolytic enzymes in sEVs. While a hypoxic phenotype was not advanced in the cisplatin-resistant NB model, our data provide direct evidence that NB-derived sEVs can reprogram the metabolome of endothelial cells in vitro.

Characterisation of the growth dynamics of HUVECs cultured in modified growth media, GF−, demonstrated a reduction in cellular viability and proliferation. Utilising this modified media, we observed an increase in tubule formation in HUVECs treated with KellyCis83 sEVs compared to Kelly sEV treatment. This suggests that the adaptation of cisplatin resistance through sEV transfer leads to enhanced anchorage-dependent differentiation without affecting proliferation or migration. Interestingly, we observed similar levels of tubule formation in HUVECs treated with KellyCis83 sEVs compared to HUVECs treated with pro-angiogenic growth media (GF+). Both Kelly and KellyCis83 sEVs had increased proliferation compared to the untreated negative control. Additionally, HUVEC migration was unaffected by either Kelly or KellyCis83 sEV treatment, which may be partially explained by the enrichment of proteins contributing to neuronal signalling. A recent study with similar results reported that prokineticin receptor 1 (PKR1) positive exosomes derived from ovarian cancer cell lines A2780 and HO-8910 increased tubule formation and migration in HUVECs but inhibited HUVEC proliferation and cell viability [46]. A tubule formation assay is a popular method for studying angiogenesis. However, our bioinformatic analysis did not detect differential abundance of pro-angiogenic proteins in KellyCis83 sEVs vs. Kelly’s sEVs; this may partially explain the modest changes in the tubule formation and warrants further investigation to determine whether these sEVs can drive angiogenic programmes.

Previous studies have demonstrated that sEVs derived from drug-resistant cancer cells promote angiogenesis via different mechanisms [47,48], although few have linked metabolic changes that promote angiogenesis and chemoresistance [15]. For example, Li et al. found that sEVs derived from cisplatin-resistant ovarian cancer cells SKOV3-DPP increased tubule formation, proliferation and migration of HUVECs in vitro, compared to sEVs derived from cisplatin-sensitive SKOV-3 cells [48]. A similar study by Zheng et al. demonstrated that EVs from 5-fluorouracil-resistant colon cancer cells (HCT-15/FU) increased HUVEC proliferation, migration and tubule formation in vitro, compared to EVs from 5-fluorouracil-sensitive HCT-15 cells [47]. In vivo, these cell-derived resistant EVs also increased microvascular density in a rat abdominal aortic neovascularisation assay, compared to EVs derived from sensitive cells. Growth/differentiation factor 15 (GDF15) was found to be enriched in HCT-15/FU EVs. When delivered to HUVECs, GDF15 increased transforming growth factor beta receptor type 3 (TGFBR-3) expression, which subsequently decreased phosphorylated SMAD2/3 expression. Inhibition of the SMAD signalling pathway in this context can increase the expression of pro-angiogenic factor periostin. Similarly, in acute myeloid leukaemia (AML), exosomes enriched with vascular endothelial growth factor (VEGF) and VEGF receptor (VEGFR) messenger RNA enhanced endothelial cell glycolysis and proliferation, leading to the promotion of chemoresistance in AML cells [15].

We also tested NB cell lines with increasing concentrations of sEVs to determine whether proliferative effects were dose-dependent. However, we found that KellyCis83’s sEVs did not transfer their proliferative phenotype to Kelly cells. Interestingly, exposure of KellyCis83 cells to 8 μg of their sEVs partially inhibited their proliferation, suggesting a potential autocrine negative feedback loop or the shedding of growth-inhibitory molecules. This intriguing observation warrants further investigation. In contrast, SH-SY5Y cells exhibited increased proliferation when treated with either Kelly or KellyCis83 sEVs in a dose-dependent manner, with no significant difference between the two. This proliferative response could be attributed to MYCN amplification in Kelly and KellyCis83 cells, an oncogene transcription factor that confers an aggressive phenotype. A study by Fonseka et al. demonstrated that sEVs from MYCN-amplified NB cells increased the colony-forming ability of non-MYCN-amplified SH-SY5Y cells [10]. In this scenario, MYCN may be able to “overwrite” some acquired changes during chemoresistance development once incorporated into sEVs [38].

While cisplatin and its derivatives remain the standard therapy for NB, patients who initially respond to treatment eventually develop chemoresistance with an approximate five-year overall survival rate of only 20%. In this study, we explored the ability of sEVs to protect cells from cisplatin cytotoxicity in vitro. However, Kelly and KellyCis83 sEVs offered equal protection against cisplatin toxicity in Kelly and SH-SY5Y recipient cells, suggesting that both share common regulators. MYCN transfer facilitated through sEVs could account for the observed limited protection against cisplatin. Notably, a similar level of ~5% protection against drugs has been reported for in vitro docetaxel-resistance in prostate cancer models, DU145RD and 22Rv1RD, despite 108- and 71-fold resistance to docetaxel compared to their respective aged parent cell lines [49]. The authors suggested that this effect was due to the transfer of MDR-1/P-gp to the recipient cells.

Recent reviews have emphasised the promise of sEVs in personalised medicine, particularly as minimally invasive liquid biopsies for diagnosis, monitoring therapy response, and predicting relapse [34,35,36]. Ongoing clinical trials exploring EV-based biomarkers reinforce their translational potential [35]. Our study reported that the MYCN oncogene was enriched in sEVs produced by NB cells with *MYCN* amplification. This provides direct evidence to support sEVs as a biomarker for detecting *MYCN* status, aiding in diagnosis, monitoring therapy response, and predicting relapse [37,38,39,50]. While our findings showed an enrichment of MYCN as a protein in sEVs, Panachan’s study reported a predictive value of exosomal-derived *MYCN* transcripts [51]. In their work, the presence and absence of exosomal *MYCN* transcripts were associated with MYCN-amplification status and treatment–relapse states [51]. Interestingly, MYCN-driven enrichment of oncogenic miRNAs (e.g., miR-16, 125b, 21, 23a, 24, 25, 27b, 218, 320a, 320b, and 92a) in exosome-like EVs was documented by [52]. Nonetheless, their study failed to confirm enrichment of EVs with MYCN. Both studies suggest that these EVs may contribute to the aggressive behaviour of *MYCN*-amplified neuroblastoma.

Furthermore, assessing the clinical relevance of some sEV proteins that contribute to glycolytic processes in conjunction with their sEV enrichment status has demonstrated an opportunity window within the context of circulating NB biomarkers. Among 13 candidates, LDHA may show great promise [53]. LDHA has been independently associated with poor outcomes in NB and proposed to be incorporated into signatures to assess the predictive power of LDHA expression to improve NB risk stratification [54]. While Dorneburg et al. evaluated LDHA expression in clinical tumour tissue samples, our study suggests assessing exosomal LDHA expression as a circulating biomarker. Therefore, sEVs may provide a strategy for developing NB progression and treatment response signatures, using less invasive methods and enabling personalised treatment approaches [55,56,57,58].

Study limitations: The role of sEVs in transferring the cisplatin resistance phenotype to cisplatin-sensitive neuroblastoma cells warrants further investigation by testing multiple NB cell lines with varying cisplatin sensitivity and longer exposure to sEVs. Expanding this concept to non-cancerous cells of TME would also deepen our understanding of sEV signalling and drug resistance development. Although the increase in cell viability after Kelly and KellyCis83 sEV treatment was statistically significant, its biological effect is relatively small, and the reported findings should be interpreted with caution, as the data were not verified in vivo models and/or clinical settings.

## 5. Conclusions

In summary, this study has highlighted a potential mechanism by which sEVs derived from cisplatin-resistant NB cells modulate various cellular effects, including metabolism, proliferation, anchorage-dependent growth of endothelial cells, and cisplatin resistance in recipient cells. Our findings demonstrated the ability of MYCN-amplified NB-derived sEVs to shift glycolysis/OXPHOS balance in NB and HUVECs, enhance tubule formation in HUVECs without proliferative or migratory effects, stimulate non-MYCN-amplified SH-SY5Y proliferation, and confer modest protection against cisplatin resistance in recipient cells. This study demonstrates a potential mechanism by which sEVs derived from cisplatin-resistant neuroblastoma cells modulate endothelial cell function through alterations in metabolic pathways and provides an opportunity to explore exosomal MYCN and glycolytic proteins for developing circulating biomarker signatures that could enable more accurate disease diagnosis and monitoring, as well as tailored treatment approaches.

## Figures and Tables

**Table 1:** HUVEC media supplementation with growth factors.

Supplement	Concentration	GF+	GF−
Foetal calf serum	0.02 mL/mL	+	+
Epidermal growth factor (EGF)	5 ng/mL	+	+
Ascorbic acid	1 μg/mL	+	+
Heparin	22.5 μg/mL	+	+
Hydrocortisone	0.2 μg/mL	+	+
Basic fibroblast growth factor (b-FGF)	10 ng/mL	+	-
Insulin-like growth factor (IGF-1)	20 ng/mL	+	-
Vascular endothelial growth factor 165 (VEGF)	0.5 ng/mL	+	-

**Table 2:** Clinical relevance of the shortlisted glycolytic candidates identified by GSEA of Kelly and KellyCis83 sEV proteome.

ID	Full Name	EFS, *p*-Value	Expression *	OS, *p*-Value	Expression	Correlation with *MYCN*	
						*r*-Value	*p*-Value
*ALDOC*	Aldolase C	2.12 × 10^−10^	⇓	1.95 × 10^−18^	⇓	−0.521	4.84 × 10^−36^
*ALPL*	Alkaline phosphatase	2.40 × 10^−5^	⇑	1.17 × 10^−5^	⇑	NS	
*ENO1*	Enolase 1	4.41 × 10^−10^	⇑	1.83 × 10^−11^	⇑	0.314	6.89 × 10^−13^
*ENO2*	Enolase 2	1.47 × 10^−10^	⇓	3.12 × 10^−18^	⇓	−0.321	1.95 × 10^−13^
*IMPA1*	Inositol(myo)-1(or 4)-monophosphatase 1	0.049	⇑	NS		−0.275	4.15 × 10^−10^
*LDHA*	Lactate dehydrogenase A	8.35 × 10^−18^	⇑	3.43 × 10^−23^	⇑	0.420	1.08 × 10^−22^
*LDHB*	Lactate dehydrogenase B	1.05 × 10^−16^	⇑	1.32 × 10^−26^	⇑	0.708	4.73 × 10^−77^
*MDH1*	Malate dehydrogenase 1	0.052	⇑	3.58 × 10^−5^	⇑	0.088	0.049
*PFKL*	Phosphofructokinase 1(liver)	4.77 × 10^−5^	⇓	6.03 × 10^−7^	⇓	NS	
*PFKM*	Phosphofructokinase (muscle)	5.32 × 10^−7^	⇑	3.34 × 10^−11^	⇑	0.557	6.67 × 10^−42^
*PGAM2*	Phosphoglycerate mutase 2	1.35 × 10^−3^	⇓	2.93 × 10^−4^	⇓	−0.244	3.72 × 10^−8^
*PKM*	Pyruvate kinase	1.85 × 10^−10^	⇑	4.62 × 10^−12^	⇑	0.299	1.02 × 10^−11^
*TPI1*	Triosephosphate isomerase 1	1.73 × 10^−11^	⇑	1.41 × 10^−10^	⇑	0.252	1.18 × 10^−8^

* Expression (high ⇑/low ⇓) that correlated with poor prognosis.

## Data Availability

The original contributions presented in this study are included in the article/Supplementary Materials. Further inquiries can be directed to the corresponding author.

## Supplementary Materials

The following supporting information can be downloaded at https://www.mdpi.com/article/10.3390/jpm15120584/s1, Figure S1: Kelly and KellyCis83 sEV have differentially expressed proteomes; Figure S2: NB cell line-derived sEVs examined with anti-MYCN antibody; Figure S3: Isolation of sEVs from the inducible SHEP-Tet-21N cell system; Table S1: Primary antibodies used for immunodetection; Table S2: List of proteins with their fold change; Table S3: Neuroblastoma cell lines and MYCN amplification status.
