# Supplementary material for: Small Extracellular Vesicles Secreted by Cisplatin-Resistant Neuroblastoma Cells Increase Lactate Secretion and Alter Metabolic Pathways in Primary Human Umbilical Vein Endothelial Cells (HUVECs)
Source: J Pers Med. 2025 Dec 1;15(12):584. doi: 10.3390/jpm15120584 (PMC12734324; doi:10.3390/jpm15120584)
Supplement: Supplementary file 1 [file jpm-15-00584-s001.zip › jpm-3853191-supplementary.pdf]

Supplementary Information

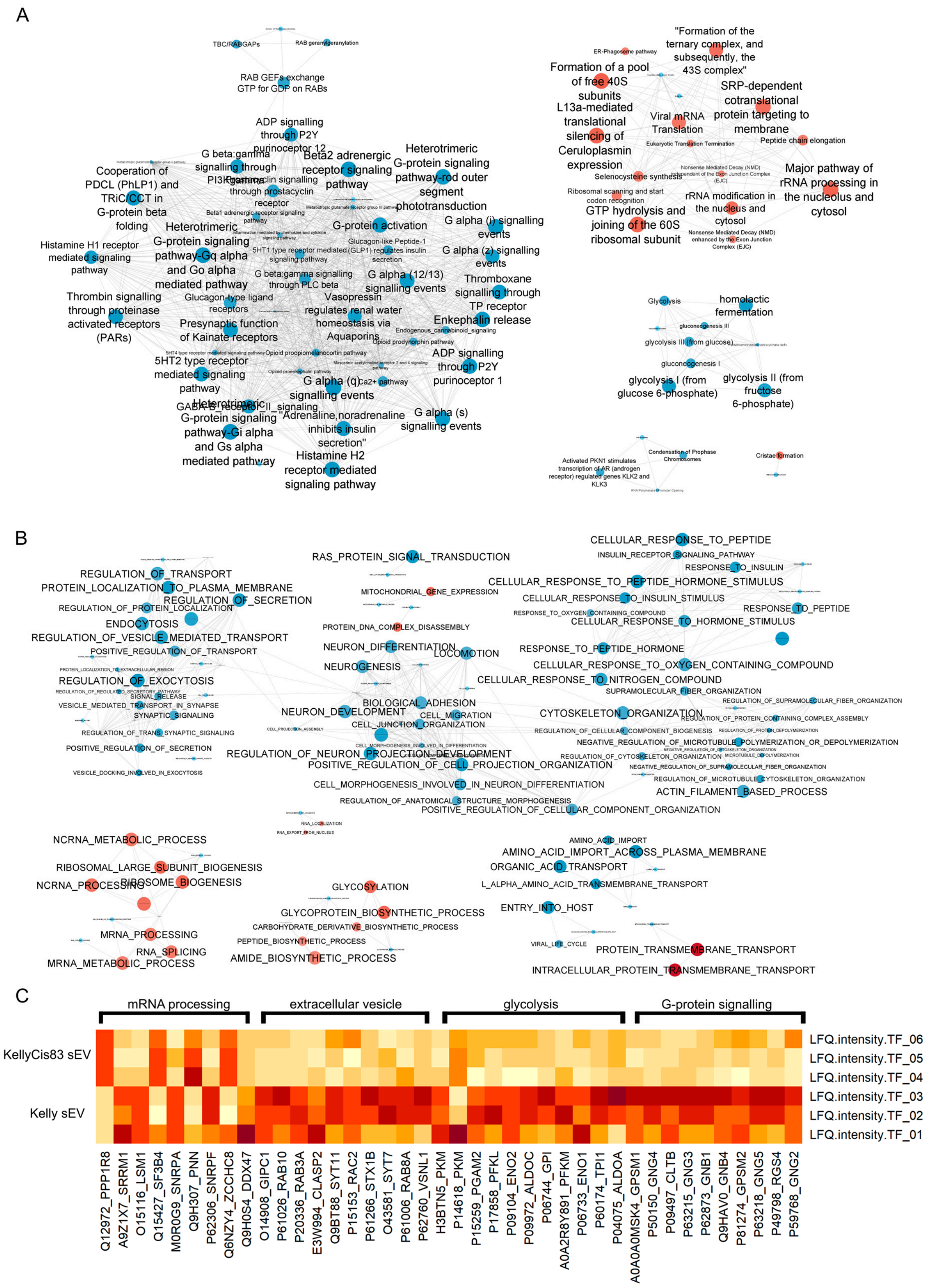

Figure S1: Kelly and KellyCis83 sEV have differentially expressed proteomes. Gene Set Enrichment Analysis using the "Pathway Commons" (A) and "Gene Ontology Biological Processes" (B) databases. A threshold of 20% overlapping proteins was used for network construction of significantly enriched pathways (adjusted p-val < 0.05). Pathways

upregulated in KellyCis83 sEV were coloured red and downregulated pathways were coloured blue. The size of the nodes was proportionally set to -log10(adjusted p-val) (C). Heatmap of normalised LFQ intensities of proteins related to mRNA processing, glycolysis, extracellular vesicle, G-protein signalling in Kelly and KellyCis83 sEV. A darker orange colour indicates a higher protein abundance.

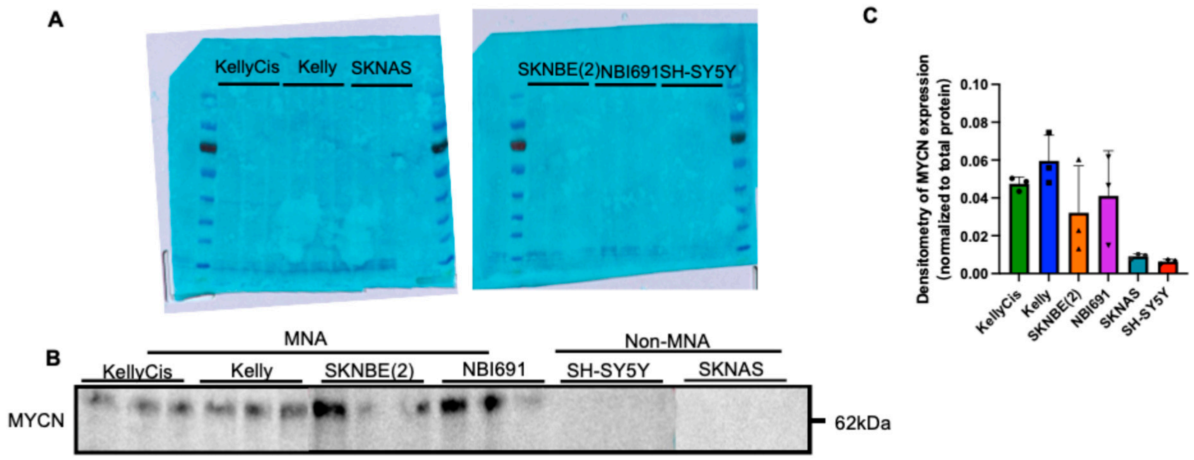

Figure S2: NB cell line-derived sEVs examined with anti-MYCN antibody. MYCN-amplified NB derived sEVs: KellyCis83, Kelly, SK-N-BE(2), and NB-1691. Non-MYCN-amplified NB derived sEVs: SH-SY5Y and SK-N-AS. (A) Total sEV protein load stained with Reversible Protein Stain Kit for Nitrocellulose (ThermoFisher, Cat no. 24580), and imaged using Amersham Imager 600 (GE Healthcare Lifescience) for protein normalization. Samples were loaded in 3 biological replicates. (B) Western blot analysis of MYCN enrichment in sEVs. (C) Densitometry analysis of MYCN detection in sEVs.

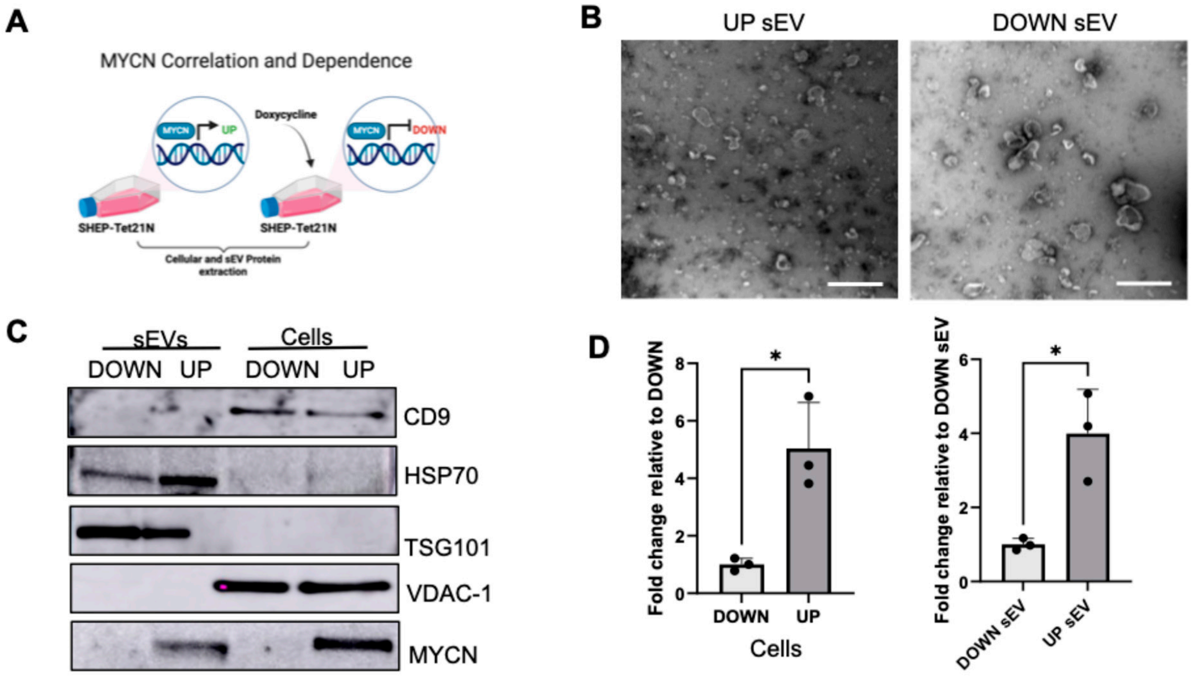

Supplementary Information

Figure S3: Isolation of sEVs from the inducible SHEP-Tet-21N cell system. (A) SHEP-Tet-21N cells were grown in the presence (MYCN-DOWN) and absence (MYCN-UP) of doxycycline, and cellular and sEV proteins were extracted for a western blot to assess whether the expression of selected glycolytic proteins is MYCN dependent. (B) TEM images of MYCN-DOWN and MYCN-UP sEV (scale bar = 500 nm); (C) Representative blot of three independent experiments, showing expression of CD9, HSP70, TSG101, and VDAC-1 in MYCN-DOWN and MYCN-UP cells and sEVs. Images for VDAC-1 and TSG101 are derived from the same blot, cut into two, prior to primary antibody incubation. The same is for CD9 and HSP70 images; (D) Densitometry analysis of MYCN in cellular and sEVs in response to doxycycline treatment . Data represented as mean ± standard deviation. Statistical significance was calculated by an unpaired t-test (* $p \leq 0.05$ and ** $p \leq 0.01$, only statistically significant values graphed). Image created with Biorender.com.

Table S1: Primary antibodies used for immunodetection

Antibody	Species	Mw (kDa)	Manufacturer	Cat no.
**EGFR**	Rb	175	Cell Signalling Technology	2963S
**GAPDH**	Ms	36	Bio-Rad, Cat no	MCA4740
**ANXA5**	Rb	30	Cell Signalling Technology	8555S
**CD9**	Rb	22, 24, 35	Cell Signalling Technology	13174S
**HSP70**	Rb	70	Cell Signalling Technology	4876S
**FLOT1**	Rb	49	Cell Signalling Technology	18634S
**TSG101**	Ms	44	Santa Cruz Biotechnology	sc-7964
**VDAC-1**	Rb	32	Cell Signalling Technology	4661S
**TUBA1A**	Rt	50	Bio-Rad	MCA78G
**SMAD4**	Rb	70	Cell Signalling Technology	38454
**LIN28B**	Rb	32, 21	Cell Signalling Technology	4196S
**LDHA**	Rb	37	Cell Signalling Technology	3582
**PKM2**	Rb	60	Cell Signalling Technology	4053
**PKM1/2**	Rb	60	Cell Signalling Technology	3990

Abbreviations: Mw = molecular weight, Rb = rabbit, Ms = mouse, Rt = rat, EGFR = epidermal growth factor receptor, GAPDH = glyceraldehyde-3-phosphate dehydrogenase, ANXA5 = annexin A5, HSP70 = heat shock protein 70, FLOT1 = flotillin, VDAC-1 = voltage-dependent anion-selective channel protein 1, TUBA1A = tubulin alpha-1A chain, SMAD4 = mothers against decapentaplegic homolog 4, LIN28B = protein lin-28 homolog B, LDHA = Lactate Dehydrogenase A, PKM1/2= Pyruvate Kinase M1/M2, PKM2= Pyruvate Kinase M2.

Supplementary Information

Table S2: List of proteins with their fold change.

**Q12972_PPP1R8**	1.41135372	0.09383154	GOBP_MRNA_PROCESSING
**A9Z1X7_SRRM1**	-1.5827254	0.16193087	GOBP_MRNA_PROCESSING
**O15116_LSM1**	-1.2154771	0.16720434	GOBP_MRNA_PROCESSING
**Q15427_SF3B4**	3.18273407	0.19337478	GOBP_MRNA_PROCESSING
**Q15427_SF3B4**	3.18273407	0.19337478	GOBP_MRNA_PROCESSING
**M0R0G9_SNRPA**	-0.9624677	0.23032851	GOBP_MRNA_PROCESSING
**Q9H307_PNN**	1.08054492	0.24146631	GOBP_MRNA_PROCESSING
**P62306_SNRPF**	-0.9908117	0.25757046	GOBP_MRNA_PROCESSING
**Q6NZY4_ZCCHC8**	2.08119875	0.2821599	GOBP_MRNA_PROCESSING
**Q9H0S4_DDX47**	-1.6054489	0.28324677	GOBP_MRNA_PROCESSING
**O14908_GIPC1**	-1.5462703	0.09354307	GOBP_REGULATION_OF_EXOCYTOSIS
**P61026_RAB10**	-1.6860884	0.16193087	GOBP_REGULATION_OF_EXOCYTOSIS
**P20336_RAB3A**	-1.1481692	0.17966354	GOBP_REGULATION_OF_EXOCYTOSIS
**E3W994_CLASP2**	-1.5254781	0.18220687	GOBP_REGULATION_OF_EXOCYTOSIS
**Q9BT88_SYT11**	-1.9633812	0.22810019	GOBP_REGULATION_OF_EXOCYTOSIS
**P15153_RAC2**	-2.1926907	0.23032851	GOBP_REGULATION_OF_EXOCYTOSIS
**P61266_STX1B**	-2.4821263	0.23999056	GOBP_REGULATION_OF_EXOCYTOSIS
**O43581_SYT7**	-2.4539054	0.26956446	GOBP_REGULATION_OF_EXOCYTOSIS
**P61006_RAB8A**	-1.1416837	0.27894719	GOBP_REGULATION_OF_EXOCYTOSIS
**P62760_VSNL1**	-2.0763637	0.31383915	GOBP_REGULATION_OF_EXOCYTOSIS
**H3BTN5_PKM**	-1.7824983	0.09354307	P00024::Glycolysis
**P15259_PGAM2**	-1.6568107	0.17966354	P00024::Glycolysis
**P17858_PFKL**	-1.5345469	0.23032851	P00024::Glycolysis
**P09104_ENO2**	-1.0807737	0.23032851	P00024::Glycolysis
**P09972_ALDOC**	-1.7563193	0.23032851	P00024::Glycolysis
**P06744_GPI**	-1.1924603	0.30110379	P00024::Glycolysis
**A0A2R8Y891_PFKM**	-0.7550127	0.32832224	P00024::Glycolysis
**P06733_ENO1**	-0.5723848	0.4165649	P00024::Glycolysis
**P60174_TPI1**	-0.5777126	0.42029655	P00024::Glycolysis
**P04075_ALDOA**	-1.0048515	0.43368964	P00024::Glycolysis
**A0A0A0MSK4_GPSM1**	-3.0966506	0.08914164	P00027::Heterotrimeric G-protein signaling pathway-Gq alpha and Go alpha mediated pathway
**P50150_GNG4**	-2.2020755	0.16193087	P00027::Heterotrimeric G-protein signaling pathway-Gq alpha and Go alpha mediated pathway
**P09497_CLTB**	-1.2261096	0.17966354	P00027::Heterotrimeric G-protein signaling pathway-Gq alpha and Go alpha mediated pathway
**P63215_GNG3**	-2.282809	0.17966354	P00027::Heterotrimeric G-protein signaling pathway-Gq alpha and Go alpha mediated pathway

**P62873_GNB1**	-1.5366645	0.19027923	P00027::Heterotrimeric G-protein signaling pathway-Gq alpha and Go alpha mediated pathway
**Q9HAV0_GNB4**	-1.5097493	0.19039491	P00027::Heterotrimeric G-protein signaling pathway-Gq alpha and Go alpha mediated pathway
**P81274_GPSM2**	-2.1536448	0.19337478	P00027::Heterotrimeric G-protein signaling pathway-Gq alpha and Go alpha mediated pathway
**P63218_GNG5**	-1.6819556	0.20675868	P00027::Heterotrimeric G-protein signaling pathway-Gq alpha and Go alpha mediated pathway
**P49798_RGS4**	-1.7940742	0.20675868	P00027::Heterotrimeric G-protein signaling pathway-Gq alpha and Go alpha mediated pathway
**P59768_GNG2**	-1.8081367	0.23032851	P00027::Heterotrimeric G-protein signaling pathway-Gq alpha and Go alpha mediated pathway

Table S3: Neuroblastoma cell lines and MYCN amplification status

Cell Line	Origin	MYCN Status
**Kelly**	1 year old female NB patient	Amplified
**KellyCis83**	Kelly-derived cisplatin-resistant cell line (Piskareva Lab)	Amplified
**NB-1691**	1 – 3 year old unspecified NB patient	Amplified
**SH-SY5Y**	4 year old female NB patient	Non-amplified
**SK-N-AS**	6 year old female NB patient	Non-amplified
**SK-N-BE(2)**	2 year, 2 month old male NB patient	Amplified
